# Severe glenohumeral ankylosis following revision reverse shoulder arthroplasty

**DOI:** 10.1016/j.xrrt.2025.100647

**Published:** 2025-12-24

**Authors:** Artem Klimov, Florian Freislederer, Alexander Pieringer, Markus Scheibel

**Affiliations:** a Department of Shoulder and Elbow Surgery, Schulthess Clinic, Zurich, Switzerland; b Center for Musculoskeletal Surgery, Charité Universitätsmedizin Berlin, Berlin, Germany

**Keywords:** Heterotopic ossification, Ankylosis, Reverse shoulder arthroplasty, Glenohumeral osteoarthritis, Joint infection, Revision surgery

Although local and small heterotopic ossification (HO) is often of mild clinical significance, more pronounced cases of HO can restrict joint mobility and leave patients unable to perform basic activities of daily living.^4^^,^^5^^,^^27^^,^^29^ HO is known to be associated with hereditary disease, central nervous system injury, burn injury, soft-tissue trauma, and surgery.^2^^,^^3^^,^^18^^,^^28^^,^^39^ The exact pathogenesis of HO remains partially unclear.^37^ Current understanding suggests that a hypoxia-adaptive microenvironment formed after trauma stimulates HO through effects of hypoxia-inducible factor-1α.^8^^,^^11^

The occurrence of HO in the glenohumeral joint was underestimated in the past.^29^ Recent studies have found HO of the glenohumeral joint to be more common than previously believed.^9^^,^^15^^,^^16^^,^^19^^,^^22^^,^^36^ The rate of HO 2-3 years after reverse shoulder arthroplasty (rTSA) has been reported to be approximately 27%.^9^^,^^22^ However, studies with longer follow-up periods have demonstrated substantially higher rates, reaching up to 75% at 10 years postoperatively.^17^^,^^19^ Usually the HO remains mild and does not lead to pronounced ossification, mostly remaining without clinical symptoms.^9^^,^^17^^,^^22^^,^^34^^,^^36^

We describe a case of periprosthetic glenohumeral ankylosis five years after rTSA in a 42-year-old patient. He had previously suffered from post-traumatic osteoarthritis following a fracture-dislocation and plate osteosynthesis of the right humeral head. Consent was obtained for the publication of case material.

## Case report

**Figure 1:**
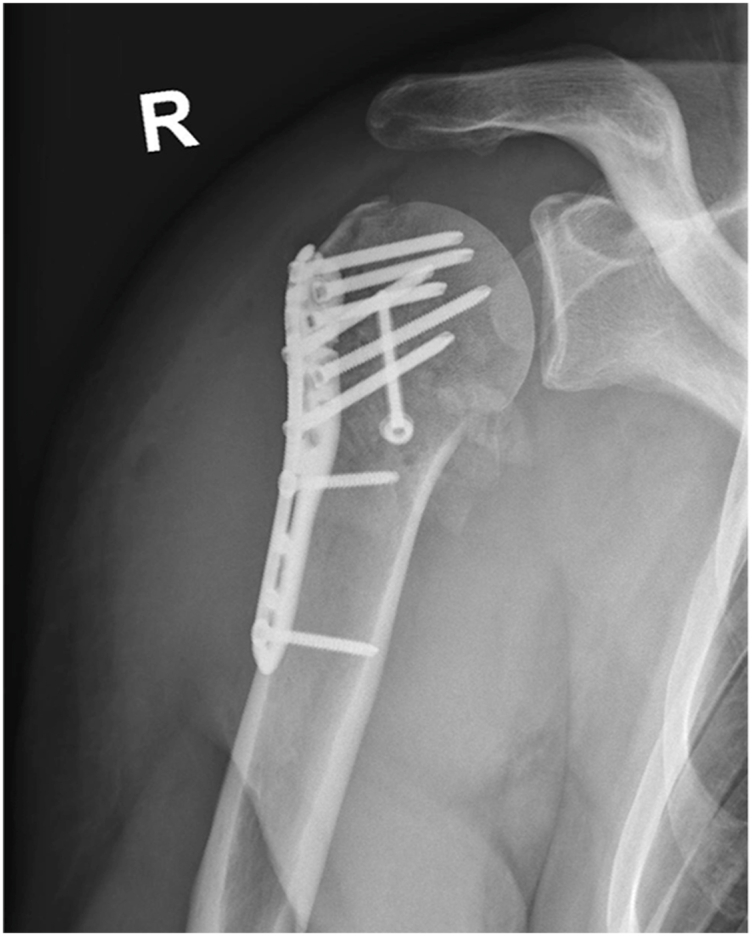
Anteroposterior radiograph of the right shoulder after plate osteosynthesis alio loco.

A 41-year-old male sustained a posterior fracture-dislocation of the right humeral head in a bicycle accident. He underwent open reduction and internal fixation with plate osteosynthesis at a local hospital (Fig. 1). The lesser tubercle was stabilized with a 3.5 mm anteroposterior cortical screw, and the shoulder was immobilized in an external rotation brace for six weeks; however, the patient discontinued brace use after 1 week due to discomfort.

Despite surgery, he reported persistent night pain during follow-ups, with minimal response to analgesics. Two months postoperatively, radiographs showed signs of delayed union. At seven months, infection was suspected due to rapid joint space narrowing, but synovial fluid cultures remained sterile. A corticosteroid injection provided no symptom relief. Due to persistent pain, progressing sintering of the humeral head, and advancing arthritic changes, arthroplasty was proposed to the patient.

One year after the accident, the patient presented at our clinic with severe pain and functional impairment of the right shoulder. He was working full-time as an engineer but had ceased all physical activity. The infectious work-up showed normal C-reactive protein and erythrocyte sedimentation rate. Physical examination of the right shoulder revealed no signs of irritation, erythema, swelling, or hyperthermia, but pronounced atrophy of surrounding muscles was noted. Active and passive flexion and abduction were limited to 30°-40° each. Internal rotation was gluteal; external rotation was not possible. The neurovascular examination was normal.

**Figure 2:**
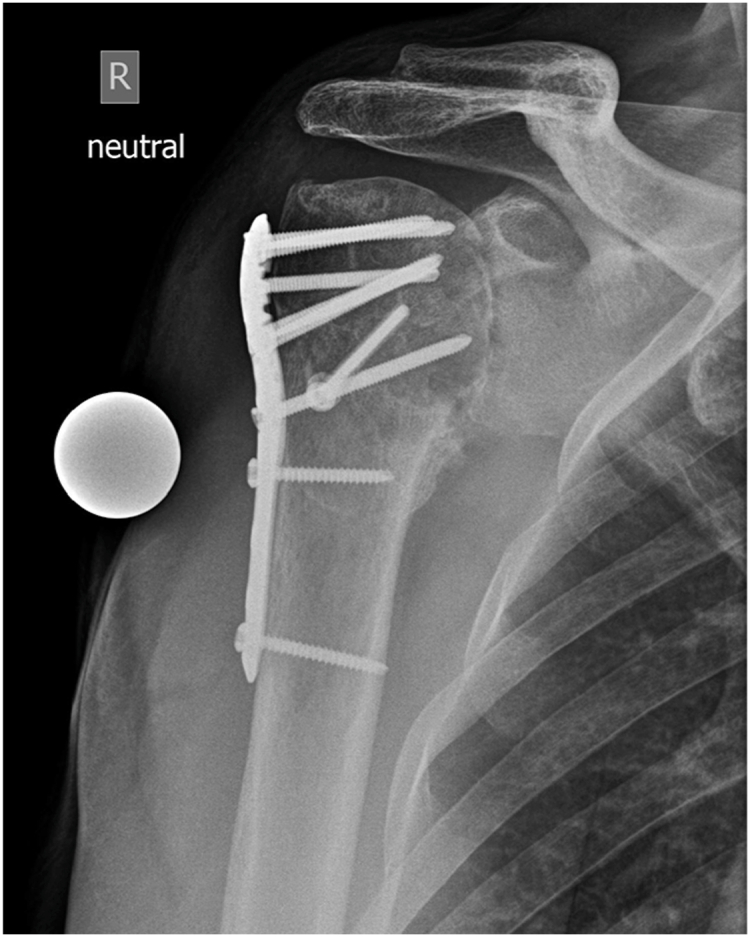
Anteroposterior radiograph of the right shoulder before revision surgery showing glenohumeral osteoarthritis and humeral osteonecrosis.

Radiographs revealed progressive sintering of the humeral head, consistent with post-traumatic osteonecrosis (Fig. 2). Computed tomography showed a consolidated fracture with intact osteosynthesis material and complete loss of glenohumeral joint space. Vast osseus defects of the humeral head and advanced osteoarthritis of the acromioclavicular joint were apparent. The acromiohumeral distance was 6 mm, glenoid retroversion measured 7°, and the rotator cuff appeared normotrophic. Due to high suspicion of low-grade infection, a two-step surgical approach was proposed with initial removal of the osteosynthesis material, collection of tissue samples, and implantation of a gentamicin-impregnated cement spacer, followed by reverse arthroplasty as a definitive treatment.

### First surgical procedure

In the beach-chair position, a standard deltopectoral approach was performed. The humeral plate was found to be extensively ingrown. A perideltoidal adhesiolysis was performed, and free suture material was removed and sent for microbiological workup. Additional tissue samples were collected from the area surrounding the plate. All screws and the plate were removed and sent for sonication. The subscapularis muscle was detached and prepared for reattachment using 4 strong polydioxanone sutures. Inspection of the humeral head revealed extensive necrotic deformations. Greasy, partially membranous tissue was observed within the drill-holes. The humeral head was resected at the anatomical neck and the metaphysis was thoroughly débrided. The supraspinatus muscle appeared very thinned and was resected. Infraspinatus and teres minor muscles were intact. A tenotomy of the biceps tendon and curettage of the glenoid were performed. After synovectomy and curettage, synovial samples were sent for microbiological and histopathological assessment. Extensive jet-lavage and débridement of the diaphysis were performed. A gentamicin-loaded cement spacer was placed on the humeral neck and the humerus was reduced. The subscapularis muscle was reattached using the preplaced polydioxanone sutures. Two Redon drains were placed, and the wound was closed. The patient received a compression bandage and a shoulder orthosis.

### First postoperative course

**Figure 3:**
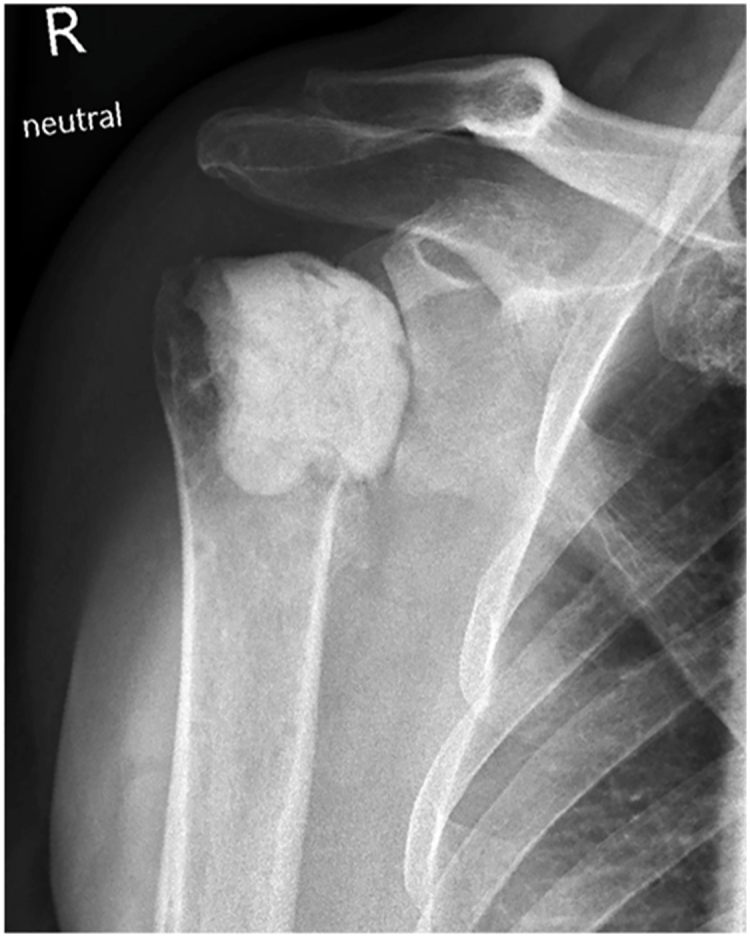
Anteroposterior radiograph of the right shoulder showing a cement spacer after the first revision surgery.

Cefuroxime was continued as an empirical perioperative antibiotic therapy. Redon drains were removed after 48 hours. Microbiological workup of collected samples revealed *Cutibacterium acnes*, *Staphylococcus capitis*, and *Staphylococcus lugdunensis*. Based on resistance profiles, the antibiotic treatment was changed to intravenous flucloxacillin and later switched to oral clindamycin until the next surgery. Neurovascular examination and inflammatory markers were normal throughout. At time of discharge from our clinic, the wound was dry and nonirritated. At the 3-week follow-up, the shoulder showed no signs of swelling or infection. Radiographs confirmed correct cement spacer positioning and no signs of HO (Fig. 3).

### Second surgical procedure

The second surgery took place after six weeks using the same deltopectoral approach. Perimembranous tissue around the spacer was excised and sent for microbiological workup. The cement spacer was removed and sent for sonication. Débridement of the shaft and excision of scarred tissue were performed, and the material also sent in. Extensive jet-lavage of the humeral spongiosa followed. A small resection in the metaphyseal area was performed to ensure good humeral anchoring of the prosthesis. A circular resection of the scarred strands around the glenoid, sparing the axillar nerve, was performed. Samples of membranous tissue from the glenoid surface were collected and sent for analysis. After extensive synovectomy and jet-lavage, the dorsal area appeared clean. To prevent dead space, the glenosphere was dorsally coated with a hemostatic collagen sponge containing gentamicin (GENTA-COLL resorb; Resorba, Nuremberg, Germany). The reverse shoulder prosthesis was inserted following standard procedures. The humeral component was cemented. Three Redon drains were placed, and the wound was closed. A compression bandage and the patient's orthosis were put on.

### Second postoperative course

The patient was started on intravenous amoxicillin/clavulanate. Redon drains were removed after 48 hours. On the third postoperative day, multiresistant *Staphylococcus epidermidis* was identified in intraoperatively collected tissue samples. The antibiotic regimen was escalated to a 12-week course of intravenous daptomycin combined with oral rifampicin. A peripherally inserted central catheter was placed for antibiotic administration. The patient remained afebrile throughout. Three weeks postoperatively, the patient was pain-free without analgesics. Five weeks postoperatively, the patient was rehospitalized with *Klebsiella pneumoniae* bacteremia associated with a central catheter-related infection and infection-associated neutropenia. He received additional intravenous antibiotics for five days, followed by a 2-week course of oral ciprofloxacin upon discharge.

**Figure 4:**
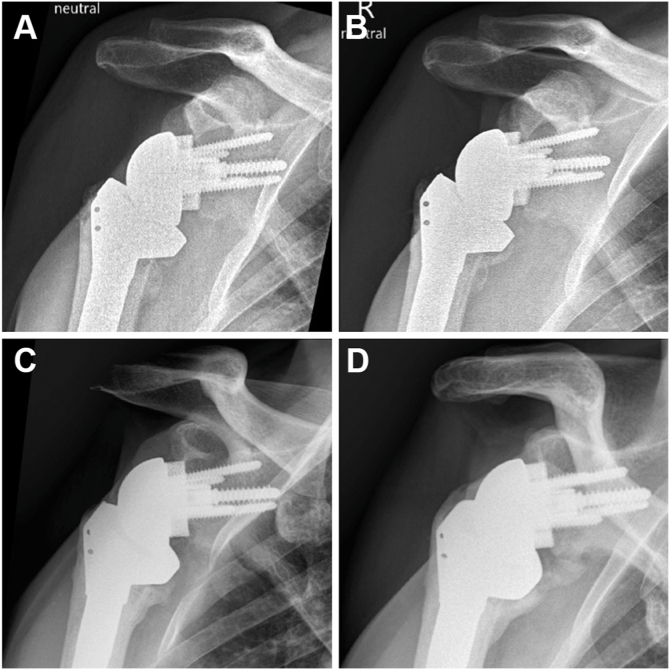
Progressive glenohumeral heterotopic ossification seen on anteroposterior radiographs of the right shoulder at (**A**) 6 weeks, (**B**) 6 months, (**C**) 1 year, and (**D**) 2 years after reverse arthroplasty.

The patient was evaluated 6 times during the following two years. His range of motion (ROM) steadily improved (Table I). All radiographs showed a centered and stable prosthesis without scapular notching (Fig. 4). Two years postoperatively, his right shoulder abduction strength was 4.5 kg vs. 8 kg on the contralateral side.

**Table I:** Range of motion testing of the right shoulder.

Follow-up	Flexion	Abduction	ER	IR
6 weeks	90°	80°	0°	Gluteal
3 mo	90°	70°	20°	Gluteal
6 mo	95°	70°	20°	
1 yr		80°		
2 yr	110°	90°		Gluteal
5 yr	140°	130°	30°	Gluteal

*ER*, external rotation; *IR*, internal rotation.

**Figure 5:**
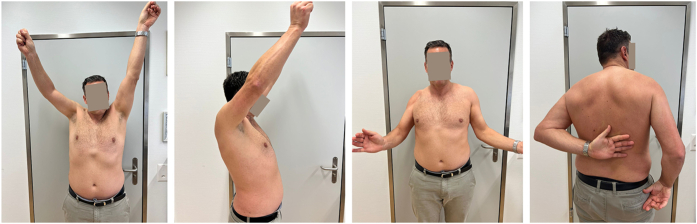
At the 5-year follow-up, the patient demonstrated good active range of motion of the right shoulder.

**Figure 6:**
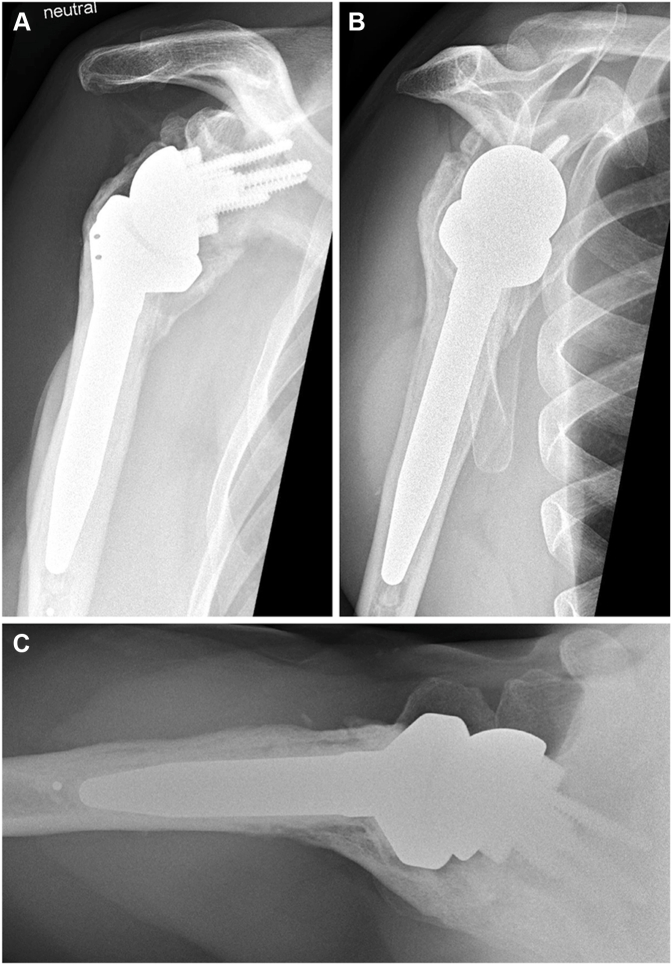
(**A**) Anteroposterior, (**B**) Neer, and (**C**) axial radiographs of the right shoulder at the 5-year follow-up showing complete glenohumeral ankylosis around the prosthesis.

At the 5-year follow-up, the patient reported no pain, good everyday function, and overall satisfaction with the outcome. He described ongoing subjective improvement in shoulder mobility. However, examination revealed no active or passive glenohumeral mobility in any direction, with all active ROM resulting from compensatory scapulothoracic motion. Despite this, functional ROM was preserved, achieving up to 140° of flexion, 130° of abduction, 30° of external rotation, and gluteal internal rotation (Fig. 5). Right shoulder radiographs revealed progressive HO, with glenohumeral bridging across all sides of the prosthesis (Fig. 6). Given the patient's functional outcome and satisfaction, no further treatment was indicated. A 10-year follow-up is planned.

## Discussion

We describe a rare case of a patient with complete glenohumeral ankylosis due to HO following revision rTSA who nevertheless achieved good everyday shoulder function and a very good functional ROM. In patients after rTSA, Verhofste et al^36^ reported a mean time of 8.3 months to form mature HO. If severe HO forms around the glenohumeral joint, patients usually present with decreasing ROM and pain.^4^^,^^7^^,^^27^^,^^29^

In a review, Zeckey et al^38^ concluded that traumatic brain injury, extensive soft tissue damage, and male sex are important patient-specific risk factors for HO. Factors proposed by other authors include repeated surgeries, difficult fracture reduction, an extensile approach, long operative time, prolonged immobilization, persistent inflammation, preexisting ectopic bone, and osteoarthritis.^10^, ^11^, ^12^^,^^23^^,^^24^^,^^30^^,^^33^^,^^35^^,^^36^ Ahrengart et al^1^ concluded that male patients with osteoarthritis or sequelae after fractures should be considered for HO prophylaxis after total hip arthroplasty.

While HO is a recognized complication following rTSA, its association with infection is less frequently reported. Kinoshita et al^14^ proposed the association of *Staphylococcus caprae* infection with severe HO after hip arthroplasty in a case report. In combat medicine, Juarez et al^12^ found infection leading to inflammation to be an important driver of HO, resulting in two-times greater odds. A review of the literature revealed no previously reported cases of infection-associated arthropathy progressing to severe HO after revision rTSA.

Our patient had a history of 2 previous surgeries, joint infections with 4 pathogens (*C. acnes*, *S. capitis*, *S. lugdunensis,* and *S. epidermidis)* leading to infection-associated arthropathy, and *K. pneumoniae* bacteremia during the postoperative course. In addition, he had several known risk factors for HO after rTSA, including male sex, prolonged immobilization, joint infection, osteoarthritis, and multiple surgeries. Retrospectively, beginning HO was visible on our patient's radiographs six weeks after surgery. The ossification increased with every follow-up, indicating unusually slow progression of HO. Due to the radiographically confirmed complete ankylosis, the patient has virtually no residual motion in the artificial joint. Nevertheless, an impressive ROM is preserved through substantially increased scapulothoracic mobility. Through this compensatory adaptation to the loss of glenohumeral motion, his ROM achieved relevant forward flexion comparable to other rTSA patients.^31^ This demonstrates the critical contribution of scapulothoracic movement to overall shoulder function.

To our knowledge, no official guidelines for prevention of glenohumeral HO exist. Studies have shown a positive effect of postoperative nonsteroidal anti-inflammatory drugs and radiation in other joints.^28^^,^^33^ Nonsteroidal anti-inflammatory drugs reduce inflammation and prevent the differentiation of mesenchymal stem cells into osteoblasts.^13^ Usually, indomethacin is used for six weeks as HO prophylaxis.^3^^,^^6^^,^^28^^,^^33^ Radiation targets osteoprogenitor cells, which proliferate and differentiate rapidly in case of HO.^3^^,^^23^^,^^32^ No significant difference has been found between effects of the 2 mentioned therapies.^3^^,^^20^^,^^21^^,^^25^ The best prophylactic treatment was achieved by combining both therapies.^26^^,^^38^ However, there is a lack of studies investigating their efficacy in preventing HO after rTSA.

There are currently no established guidelines for the treatment of ankylosis after rTSA. Literature indicates that once HO has formed, surgical resection is the only effective treatment.^11^^,^^32^ Our patient is satisfied with the current situation and demonstrates remarkably good ROM, considering the clinical circumstances. As in the saying, “treat the patient, not the radiograph,” we will proceed with our standard follow-up plan.

## Conclusion

To our knowledge, this is the first documented case of a patient with a completely ankylosed glenohumeral joint due to HO following revision rTSA who reports good everyday shoulder function and shows a very good ROM due to increased scapulothoracic mobility. The unusually slow appearance of HO together with intensive physiotherapy might have contributed to this unexpected outcome. It should be noted that the probability of postoperative HO is increased by male sex, repeated surgeries, extensive soft tissue damage, long-lasting infection, and osteoarthritis following a fracture. In patients at risk, postoperative HO prophylaxis after rTSA should be considered. This case highlights that even in the presence of a radiographically confirmed ankylosis, a more than satisfactory outcome is possible without the need for revision surgery.

## Disclaimers

Funding: No funding was disclosed by the authors.

Conflicts of interest: Florian Freislederer is a consultant for Stryker. Markus Scheibel is a consultant for Stryker. The other authors, their immediate families, and any research foundation with which they are affiliated have not received any financial payments or other benefits from any commercial entity related to the subject of this article.

Patient consent: Obtained.
